# Machine learning-based prediction of activities of daily living in patients with stroke and other acquired brain injuries

**DOI:** 10.1097/MD.0000000000047811

**Published:** 2026-02-20

**Authors:** Chizuko Omi, Yuichi Wada

**Affiliations:** a Department of Recovery Support, Tohoku-kai Hospital, Sendai, Miyagi, Japan; b Department of Cognitive Psychology and Informatics, Graduate School of Information Sciences, Tohoku University, Sendai, Miyagi, Japan.

**Keywords:** acquired brain injury, activities of daily living, Functional Independence Measure, machine learning, rehabilitation outcomes, rehabilitation prediction, stroke

## Abstract

In rehabilitation research, machine learning (ML) algorithms are being increasingly applied to predict the ability of patients with stroke and other acquired brain injuries to perform activities of daily living (ADL). However, many previous studies have relied on complex data, such as imaging or blood tests, which require advanced equipment and specialized expertise and are difficult to apply in general clinical settings. We aimed to predict ADL independence in patients with stroke and other acquired brain injuries at the start of rehabilitation using routine assessment data. This retrospective study involved 379 patients with stroke or other acquired brain injuries who received rehabilitation at our hospital between September 2008 and August 2022. Three ML models – support vector machine (SVM), random forest (RF), and Light Gradient-Boosting Machine (LightGBM) – were used to classify Functional Independence Measure scores. Predictors included age, sex, Japan Coma Scale score, and Brunnstrom recovery stage. All models performed above chance, with SVM achieving the highest F1 score (0.862), followed by RF (0.857) and LightGBM (0.838). Analysis of variance revealed a significant effect of the classifier on performance (*P* < .001), with SVM and RF significantly outperforming LightGBM. SHapley Additive exPlanations analysis identified lower- and upper-limb Brunnstrom recovery stage, impaired consciousness, and age as key predictive features. ML algorithms can accurately predict ADL independence using initial rehabilitation assessment data without reliance on advanced medical technologies. The use of these algorithms may enhance clinical decision-making, and they may be particularly valuable in resource-limited settings.

## 1. Introduction

Stroke remains the second leading cause of death and the third leading cause of death and disability combined worldwide, accounting for approximately 7.3 million deaths and 160 million disability-adjusted life years in 2021. Globally, there were an estimated 93.8 million prevalent cases and 11.9 million incident strokes that year. As a major cause of acquired brain injury (ABI), stroke frequently results in long-term physical, cognitive, and psychosocial impairments that restrict independence and activities of daily living (ADL), even among survivors. Despite improvements in prevention and acute care, the global stroke burden continues to rise, particularly in low- and middle-income countries (GBD 2021, Stroke Collaborators 2024). Accurately predicting physical and cognitive outcomes, including ADL independence, is therefore essential for planning and implementing individualized rehabilitation strategies, particularly in patients with stroke or other ABIs.

Functional Independence Measure (FIM) is widely used to assess ADL following stroke and other ABIs because it provides a comprehensive evaluation of both physical functions (e.g., feeding, toileting, and mobility) and cognitive functions (e.g., memory and social interaction).^[1]^ Stroke and other ABIs, including traumatic brain injury and hypoxic brain injury, often result in similar motor paralysis and higher-level brain dysfunction, leading to shared rehabilitation goals and the use of standardized outcome measures such as the modified Rankin Scale (mRS), Barthel Index, and FIM.

Estimating the total FIM score, which reflects the overall level of ADL independence, is an important component of early rehabilitation planning.

Previous studies predicting ADL independence have primarily used regression analysis to predict indicators, such as the mRS, Barthel Index, and FIM.^[2–4]^ However, in recent years, ML algorithms have increasingly been used in healthcare and rehabilitation to predict ADL independence and prognosis. Specifically, there has been active research using the mRS as a predictor of functional recovery after stroke and other ABI. For example, one study performed a binary classification of mRS scores after vascular recanalization treatment.^[5]^ Another study used binary classification of mRS to predict 6-month survival after stroke.^[6]^ However, estimating the FIM total score, which reflects the overall level of ADL independence, is an important component of early rehabilitation planning, yet research focusing on such predictive models remains limited.

Most previous studies have used complex features, such as biochemical markers and neuroimaging data, which are difficult to obtain during initial rehabilitation.^[7–10]^ This approach is suitable for facilities with advanced equipment but is not feasible in general clinical settings. Furthermore, although several studies have predicted patients' ADL performance at 90 days or 6 months after stroke,^[11–14]^ to our knowledge, no studies have focused on predicting patients' ADL performance at the start of rehabilitation.

Predicting overall ADL independence using clinical variables obtained from initial rehabilitation assessments offers many advantages, including the development of individualized treatment and rehabilitation strategies and environmental adjustments. This approach is particularly advantageous for rehabilitation in hospitals and facilities without advanced medical equipment.

Previous studies have used an FIM score of 90 or higher as a practical and clinically meaningful criterion rather than a statistically optimized cutoff.^[15–17]^ Giaquinto et al defined an FIM score > 90 as a good recovery, and Luk et al similarly defined an FIM score ≥ 90 as a satisfactory rehabilitation outcome. Expanding on this clinical practice, Sato et al empirically derived an FIM cutoff value for home discharge depending on the number of family caregivers, reporting a value of 30%.^[17]^

Against this background, we developed an ML model to predict ADL performance at the start of rehabilitation in patients with stroke or other ABI using readily available data on clinical variables, such as gender, motor paralysis, and severity of consciousness impairment. Specifically, we created a binary classification model to predict an FIM score of 90 or higher using readily available data on clinical variables, such as gender, motor paralysis, and severity of consciousness impairment, and developed an ML model to predict ADL performance at the start of rehabilitation in patients with stroke or other ABI.

## 2. Methods

### 2.1. Data sources and study participants

In this retrospective study, we analyzed clinical data retrieved from the medical records of patients with stroke and other ABIs, including cerebral infarction, cerebral hemorrhage, subarachnoid hemorrhage, traumatic brain injury, brain tumor, and encephalitis, who were admitted to or treated at our hospital between September 2008 and August 2022. The patients provided written informed consent for the use of their medical information for research purposes. The exclusion criteria included missing data regarding age, sex, Brunnstrom recovery stage (BRS), Japan Coma Scale (JCS) score, or FIM score. Data were collected at the initiation of inpatient and outpatient rehabilitation. The final cohort size comprised 379 patients. This study was approved by the Human Research Ethics Committee of the Graduate School of Information Sciences, Tohoku University (Protocol No. 23A-03).

The predictor variables were age, sex, BRS, and JCS score. Information on age and sex was retrospectively collected from medical records. Occupational or physical therapists assessed FIM, BRS, and JCS scores at the time of admission or at the initiation of outpatient rehabilitation. For predictor variables, excluding age and sex, a standard scaler was fit to the training data, and both the training and test samples were standardized to have a mean of 0 and a standard deviation of 1.

### 2.2. Data analysis

We developed predictive models using a set of 6 features to reliably estimate the FIM of the participants. Outcomes were assessed using the FIM scores, with a cutoff of ≥90 to classify patients as requiring supervision (FIM ≥ 90) or assistance (FIM < 90). The classification accuracies of the ML models were evaluated. We used 3 ML models (or classifiers), namely, random forest (RF), support vector machine (SVM), and Light Gradient-Boosting Machine (LightGBM). We implemented all models using Python 3.11.8 (Python Software Foundation, Wilmington). The ML models, including RF and LightGBM, were built using the scikit-learn (version 1.6.1) and LightGBM (version 4.5.0) libraries, respectively. Additionally, we used NumPy 2.1.0 (NumPy Developers) and pandas 2.2.3 (NumFOCUS, Austin) for data manipulation.

Although the theoretical chance level for classification should be 0.5, an unbalanced number of samples in each class can cause slight deviations from this level under random prediction. To address this issue, we compared our classifiers with those of a baseline model to determine the significance of the observed accuracy levels. Specifically, we used a dummy classifier with a “stratified strategy” implemented in scikit-learn, which generates predictions by randomly respecting the class distribution of the training set. This comparison allowed us to assess whether the accuracy of our classifiers was substantial compared with that of the dummy classifier.

The macro-averaged F1 score, defined as the harmonic mean of precision and sensitivity, was calculated as the primary metric for evaluating the performance of the classifiers. Precision for a class indicates the proportion of true positives among predicted instances, whereas sensitivity (recall) indicates the proportion of true positives correctly identified by the classifier out of all actual instances. The macro-averaged F1 score was computed using the arithmetic mean of the per-class F1 scores. This approach prevents a classifier from achieving high scores by favoring more frequent categories, offering an advantage over relying solely on accuracy. To obtain unbiased macro-averaged F1 scores, the classification models were evaluated using a nested (outer: 5-fold; inner: 5-fold) cross-validation procedure with Stratified *K*-Fold in scikit-learn. Hyperparameters were selected based on the F1 score in the inner loops, and F1 scores were collected in the outer loops. This nested cross-validation cycle ensured that data from all participants appeared only in the training or validation datasets. The Stratified *K*-Fold cross-validation procedure ensured an equal number of samples across the 5-folds. To enhance classification stability, the process was repeated 20 times with different training and testing splits, and the average estimate of the F1 scores and the 95% confidence interval (CI) around the mean, computed from the set of 20 F1 scores for each classifier, were reported. To investigate the feasibility of accurately classifying patients into those requiring supervision (FIM ≥ 90) or assistance (FIM < 90), a one-way repeated-measures analysis of variance (ANOVA) was conducted with the F1 score serving as the dependent variable and classifier (RF, SVM, and LightGBM) as the independent variable. Greenhouse–Geisser corrections were applied when the assumption of sphericity was violated. Given multiple ANOVAs and subsequent pairwise comparisons, the reported *P*-values were corrected using the Bonferroni–Holm procedure.

## 3. Results

The mean age of the patients was 79.0 ± 10.87 years (median, 82 years; mode, 84 years). The youngest participant was 35 years old, and the oldest was 99 years old, with most patients being older adults.

The baseline characteristics of the supervision (FIM ≥ 90) and assistance-required (FIM < 90) groups are shown in Table 1. There were 191 male patients (51.7%) and 178 female patients (48.3%), with no significant sex difference. However, there were more female patients in the assistance-required group and more male patients in the supervision group (Fisher exact test, *P* = .059).

**Table 1:** Baseline characteristics of the supervision (FIM **≥** 90) and assistance-required (FIM **<** 90) groups.

	<90	≥90	*P*-value
Age (yr)	82.4 (±8.6)	71.7 (±12.6)	<.001
Sex ratio	127/134	70/48	–
Upper-limb BRS	3.36 (±1.6)	5.68 (±0.7)	<.001
Fingers BRS	3.64 (±1.7)	5.66 (±0.7)	<.001
Lower-limb BRS	3.67 (±1.5)	5.72 (±0.5)	<.001
JCS	2.33 (±2.3)	0.33 (±0.2)	<.001
FIM	37.4 (±21.6)	108.5 (±11.1)	<.001

The numbers in parentheses indicate the standard deviation.

BRS = Brunnstrom recovery stage, FIM = Functional Independent Measure, JCS = Japan coma scale.

We compared age, BRS, JCS scores, and FIM scores between the groups using a Welch *t* test. Participants in the assistance-required group were significantly older on average and had lower BRS, JCS scores, and FIM scores than those in the supervision group.

Table 1 shows the baseline characteristics of the 2 groups: those with FIM ≥ 90 and those with FIM < 90. The distributions of consciousness level and motor function among all participants are presented in Appendix Figures 1 to 4 (Supplemental Digital Content, https://links.lww.com/MD/R439). In the FIM ≥ 90 group, the mean JCS score was 2.33, indicating mild impairment of consciousness, whereas in the FIM < 90 group, the mean JCS score was 0.33.

For all participants combined, the JCS distribution was as follows (Appendix Figure 1, Supplemental Digital Content, https://links.lww.com/MD/R439): 0 = 210, I-1 = 11, I-2 = 21, I-3 = 52, II-10 = 18, II-20 = 39, II-30 = 10, III-100 = 0, III-200 = 0, and III-300 = 9. Overall, while impaired consciousness was present in some participants, it was generally mild in severity. The distributions of BRS are presented in Appendix Figures 2 to 4, Supplemental Digital Content, https://links.lww.com/MD/R439. For the upper limb, Appendix Figure 2, Supplemental Digital Content, https://links.lww.com/MD/R439, the distribution was as follows: I = 14 (3%), II = 88 (23%), III = 32 (8%), IV = 41 (10%), V = 52 (13%), and VI = 152 (40%).

For the hand, Appendix Figure 3, Supplemental Digital Content, https://links.lww.com/MD/R439, the distribution was as follows: I = 23 (6%), II = 85 (22%), III = 21 (5%), IV = 44 (11%), V = 49 (12%), and VI = 157 (41%). For the lower limb, Appendix Figure 4, Supplemental Digital Content, https://links.lww.com/MD/R439, the distribution was as follows: I = 7 (1%), II = 81 (21%), III = 43 (11%), IV = 42 (11%), V = 66 (17%), and VI = 140 (36%). Approximately half of the participants had mild motor paralysis in the upper limb, hand, and lower limb.

The average F1 scores and corresponding 95% CIs for each ML-based model are summarized in Table 2. Participants were classified at levels well above chance in all 3 classifiers, with nonoverlapping 95% CIs, indicating significantly higher classification accuracy than chance. The SVM classifier yielded the best F1 score of 0.862, whereas RF and LightGBM yielded F1 scores of 0.857 and 0.838, respectively. To evaluate the classification performance of each model, we conducted a series of one-way repeated-measures ANOVAs with a classifier (RF, SVM, LightGBM) as the independent variable and F1 score as the dependent variable. There was a significant main effect of the classifier on the F1 score (*F* [2,38] = 43.1, *P* < .001, *η*_*p*_^2^ = .694). Post hoc pairwise comparisons revealed that the RF and SVM models outperformed the LightGBM model (*P* < .001), with no difference in the F1 scores between the RF and SVM models.

**Table 2:** Average F1 scores for each model in the outer validation set.

Model	F1 score	SD	95% CI
Baseline	0.497	0.024	(0.486–0.509)
RF	0.858	0.008	(0.854–0.862)
SVM	0.862	0.009	(0.858–0.867)
LightGBM	0.839	0.011	(0.834–0.844)

CI = confidence interval, LightGBM = Light Gradient-Boosting Machine, RF = random forest, SD = standard deviation, SVM = support vector machine.

**Figure 1.**
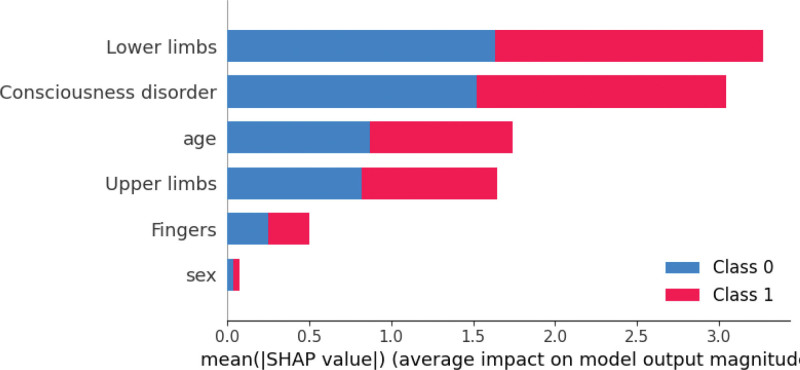
Summary plot for SHAP analysis showing the mean absolute SHAP value of the features in predicting the ability of patients to perform ADL. Class 0 corresponds to the class of patients requiring assistance (FIM < 90), and class 1 corresponds to those requiring supervision (FIM ≥ 90). ADL = activities of daily living, FIM = Functional Independence Measure, SHAP = SHapley Additive exPlanations.

To determine the features contributing most to the classification results, we performed SHapley Additive exPlanations (SHAP) analysis of the SVM results. The SHAP values provided insights into the contribution of each feature to the model predictions. Figure 1 displays a feature importance graph based on SHAP values, with key features listed at the top. BRS of the lower limb was the most significant feature, followed by impaired consciousness, age, and upper-limb BRS.

**Figure 2.**
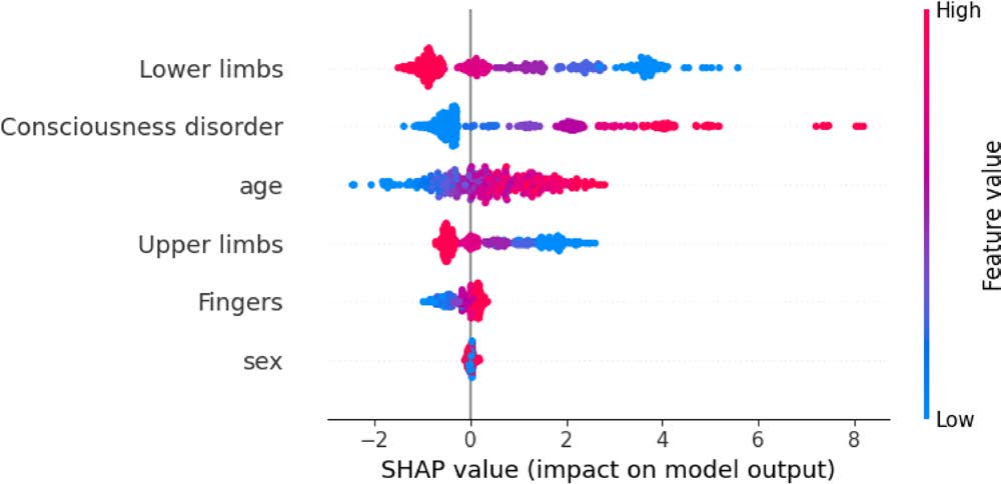
Beeswarm plot for SHAP analysis showing the features in the SVM model contributing to predicting the ability of patients to perform ADL. The plot colors represent feature value levels, with red indicating positive contributions to the assistance-required group and blue indicating negative contributions. ADL = activities of daily living, SHAP = SHapley Additive exPlanations, SVM = support vector machine.

To explore how each feature influenced classification into the supervision and assistance-required groups, we generated a beeswarm plot (Fig. 2). The horizontal axis shows the SHAP values, while the vertical axis lists features in descending order of importance. Each feature plot is spread horizontally, indicating the absolute SHAP value and its contribution to classification, with red indicating positive and blue indicating negative contributions to classification into the assistance-required group. A lower BRS for the limbs and higher values for age and impaired consciousness positively affected classification into the assistance-required group (Fig. 2).

## 4. Discussion

In this study, we estimated FIM scores at the start of rehabilitation in patients with stroke and other ABIs using ML models. The 3 models, RF, SVM, and LightGBM, achieved higher F1 scores than the baseline model. The key features in estimating FIM scores were the BRS of the lower limbs, the JCS score, and age. Otieno et al^[18]^ developed ML models using nationwide registry data from Sweden to predict the mRS in 3 categories of patients: independent, dependent, and deceased. Their study demonstrated that artificial neural networks and eXtreme Gradient Boosting performed significantly better than multinomial logistic regression in classifying functional dependence. Specifically, the F1 scores were 0.603 (95% CI = 0.594–0.611) and 0.577 (95% CI = 0.568–0.586) for artificial neural networks and eXtreme Gradient Boosting, respectively, and 0.544 (95% CI = 0.545–0.563) for multinomial logistic regression. By contrast, our study focused on the binary classification of FIM-based functional independence and dependence, in which the SVM model achieved the highest F1 score (0.862), followed by RF (0.857) and LightGBM (0.838). These results indicate higher predictive performance than that reported by Otieno et al.^[18]^

One of the key characteristics of this study was the use of simple clinical variables, such as age, JCS score, and BRS, which can be assessed during the initial rehabilitation evaluation. Previous research has shown that age, medical history, bowel and bladder control, consciousness disorders, orientation disorders, severity of paralysis, level of social support, and sitting balance ability affect recovery in ADL performance.^[19]^ Additionally, patients with consciousness disorders have poor functional prognoses.^[20]^ Lower-limb BRS and walking and balance abilities are correlated with physical function recovery.^[21]^ Our results are consistent with these previous findings. In rehabilitation, consciousness disorders hinder voluntary physical movement, making functional training less effective.^[22]^ Severe flaccid paralysis also challenges the recovery of motor function and ADL performance.^[23]^ These factors contributed to the high importance of these variables in estimating FIM in this study. Conversely, sex and upper-limb BRS had limited significance in the binary classification based on FIM. In this study, there were more male patients in the independent group, and more female patients in the dependent group, reflecting the finding of previous research, that is, female patients tend to have lower ADL independence poststroke.^[24]^ Despite skewed age and FIM score distributions, we found similar FIM scores between sexes. Future research should investigate whether similar results are obtained after adjusting for age and FIM score distribution. The low importance of the upper-limb BRS may be due to its compensation with assistive devices or the involvement of the nonparalyzed side during ADL, minimizing its impact on ADL independence and predictive importance.

A strength of this study was that simple clinical variables, obtained through interviews and behavioral observations during the initial rehabilitation period, were used to predict the ability to perform ADL without using any advanced medical equipment. This approach may help medical professionals develop tailored treatment plans and perform environmental adjustments.

In this study, the number of scores below the FIM cutoff value was large. Therefore, we used the F1 score to mitigate the effect of imbalanced data. Our classification accuracy was slightly lower than that in a previous study with over 40,284 participants, which is > 10 times the size of our cohort,^[25]^ but higher than that of a previous study with 499 participants.^[6]^ In ML, larger datasets typically improve accuracy, and the results of the larger study suggest that abundant training data enhance model performance. Despite using a smaller, imbalanced dataset, we constructed a model with high clinical applicability.

This study had several limitations. First, it used data from a single facility with a cohort predominantly comprising older individuals and a total FIM score of <90, indicating low ADL independence. Second, the model was constructed using a relatively small cohort of 379 participants. Future research should examine whether similar results can be obtained using data from multiple facilities, broader age groups and ADL levels, and larger datasets. Third, we included participants with stroke and other acquired brain injuries who exhibited similar motor paralysis, cognitive impairment, and reduced ADL ability. Future studies should assess whether comparable results can be obtained when analyzing disease-specific data, such as data on patients with cerebral infarction, cerebral hemorrhage, subarachnoid hemorrhage, and traumatic brain injuries. Fourth, although we used the BRS score as an indicator of motor paralysis, additional assessments such as the Fugl–Meyer assessment and 12-step hemiplegic functional assessment should be considered in future ADL prediction and classification research. Finally, factors such as hemispatial neglect and sensory impairments,^[26,27]^ which were not included in this study, should also be considered for their potential contributions to ADL index classification.

In addition, we did not include potentially important factors, such as patient comorbidities, lesion size or location, length of hospital stay, family or caregiver support, type and frequency of rehabilitation therapies, or patients' socioeconomic status or income. Considering these factors could provide a more comprehensive understanding of the determinants of ADL independence and improve the generalizability of the findings.

However, the primary aim of this study was to develop a screening model for ADL ability at the initiation of rehabilitation. Therefore, we intentionally limited the predictors to a minimal set of variables that can be readily obtained at the very beginning of rehabilitation, without requiring advanced imaging, specialized interpretation, or time-consuming data collection. This design was intended to ensure clinical applicability and feasibility in real-world rehabilitation settings.

In future research, models incorporating additional variables – such as comorbidities, lesion characteristics, rehabilitation details, and social factors – will be developed and compared with the present model using data collected after a certain period of rehabilitation. Such an approach may allow for a more in-depth understanding of ADL prognosis and contribute to the development of more individualized and adaptive rehabilitation strategies for patients with stroke and other ABIs.

In conclusion, the SVM, RF, and LightGBM models effectively predicted ADL independence at the start of rehabilitation using initial assessment data. Our findings suggest that ADL independence can be accurately predicted without using any advanced medical equipment, thereby facilitating broader clinical implementation of our approach.

## Acknowledgments

We appreciate the staff of Katakura Hospital for their cooperation with data collection. A part of this work was presented at the 8th Annual Autumn Meeting of the Japanese Association of Rehabilitation Medicine.

## Author contributions

**Conceptualization:** Chizuko Omi.

**Data curation:** Chizuko Omi.

**Formal analysis:** Chizuko Omi.

**Methodology:** Chizuko Omi.

**Project administration:** Chizuko Omi.

**Validation:** Chizuko Omi.

**Visualization:** Chizuko Omi.

**Supervision:** Yuichi Wada.

**Writing – original draft:** Chizuko Omi.

**Writing – review & editing:** Chizuko Omi.

## Supplementary Material
