# Supplementary material for: Machine learning-based prediction of activities of daily living in patients with stroke and other acquired brain injuries
Source: Medicine (Baltimore). 2026 Feb 20;105(8):e47811. doi: 10.1097/MD.0000000000047811 (PMC12928916; doi:10.1097/MD.0000000000047811)
Supplement: Supplementary file 1 [file medi-105-e47811-s001.pdf]

Appendix1

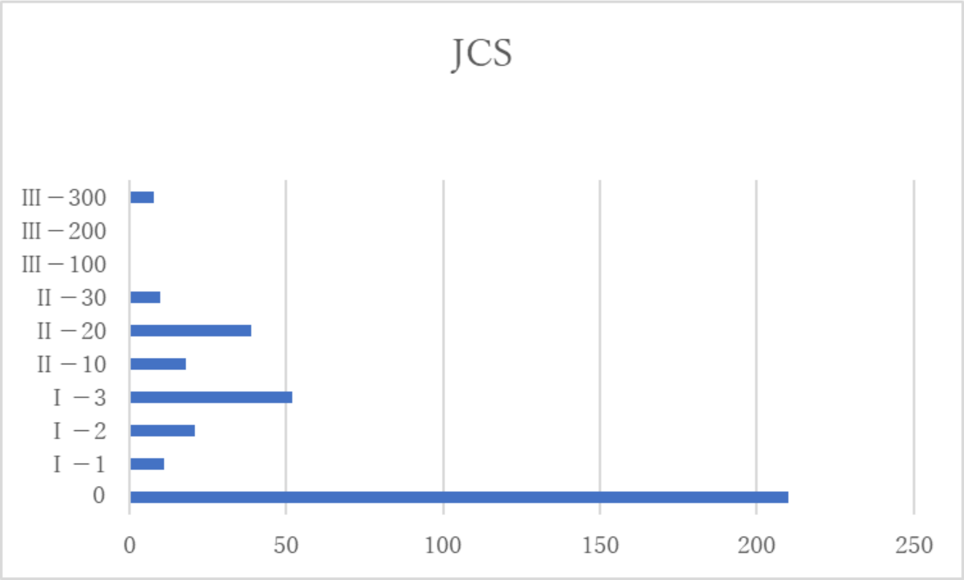

Figure 1. Distribution of the Japan Coma Scale (JCS) scores.

Appendix2

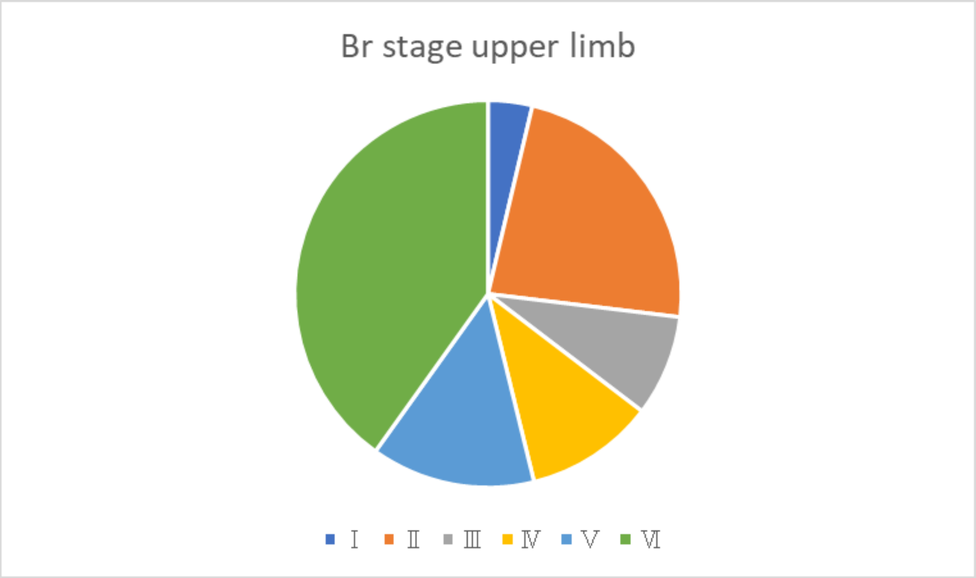

Figure 2. Distribution of Brunnstrom Recovery Stage (BRS) for the upper limb.

Appendix3

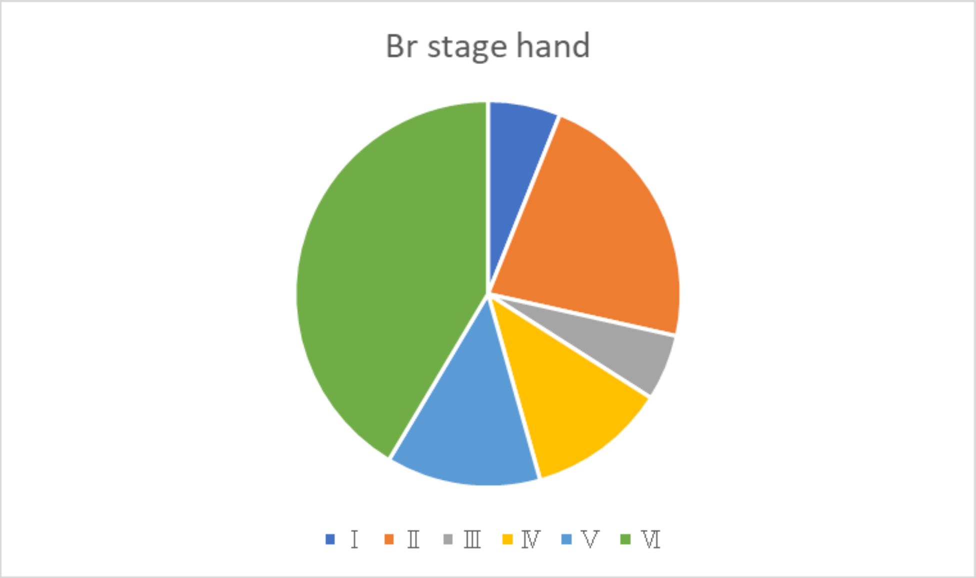

Figure 3. Distribution of Brunnstrom Recovery Stage (BRS) for the hand.

Appendix4

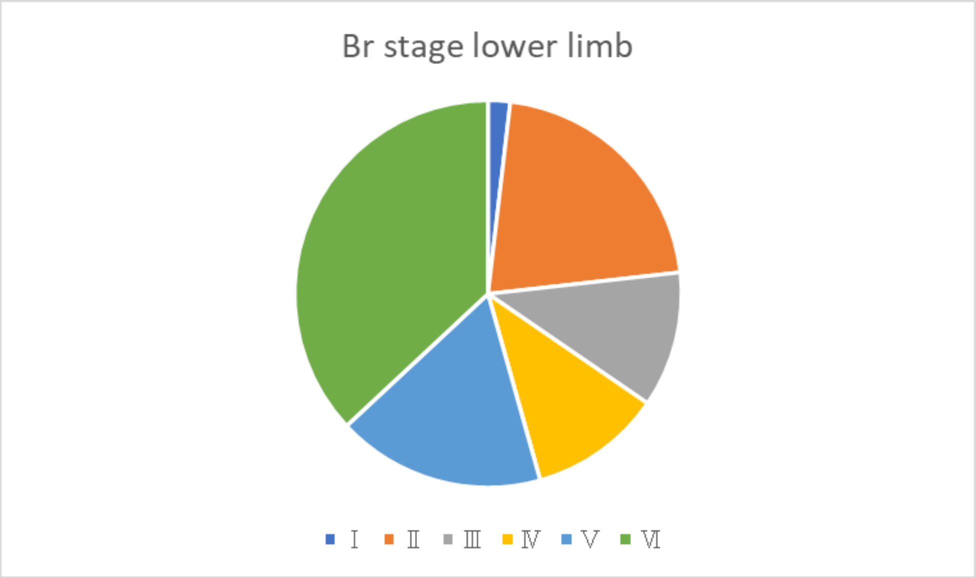

Figure 4. Distribution of Brunnstrom Recovery Stage (BRS) for the lower limb.
